# Supplementary material for: Dynamics of Staphylococcus aureus Cas9 in DNA target Association and Dissociation
Source: EMBO Rep. 2020 Aug 13;21(10):e50184. doi: 10.15252/embr.202050184 (PMC7534634; doi:10.15252/embr.202050184)
Supplement: Supplementary file 1 — Appendix [file EMBR-21-e50184-s001.pdf]

TABLE OF CONTENTS

Appendix Fig S1 - Positions and disruption forces of the two interactions between dSaCas9/sgRNA-2 and its DNA target. 2

Appendix Fig S2 - Positions and disruption forces of the two interactions between dSaCas9 and DNA when guided by partially matched sgRNAs. 3

Appendix Fig S3 - DNA cleavage by SaCas9 when guided by PAM-distal mismatched sgRNAs. 4

Appendix Fig S4 - Confocal image of the λ DNA in the presence of crRNA or crRNA:tracrRNA.. 5

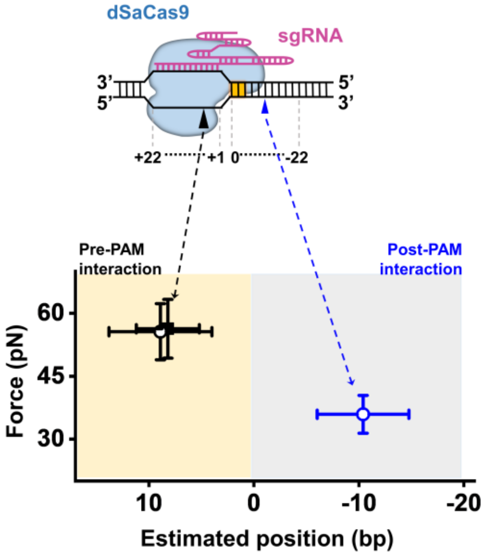

Appendix Fig S1 - Positions and disruption forces of the two interactions between dSaCas9/sgRNA-2 and its DNA target.

Positions and disruption forces of interactions between dSaCas9/sgRNA-2 and its DNA target obtained in the forward (n = 15) and reverse (n = 20) DNA unzipping experiments. The pre- and post-PAM interactions are indicated in black and blue, respectively. The error bars represent the SD.

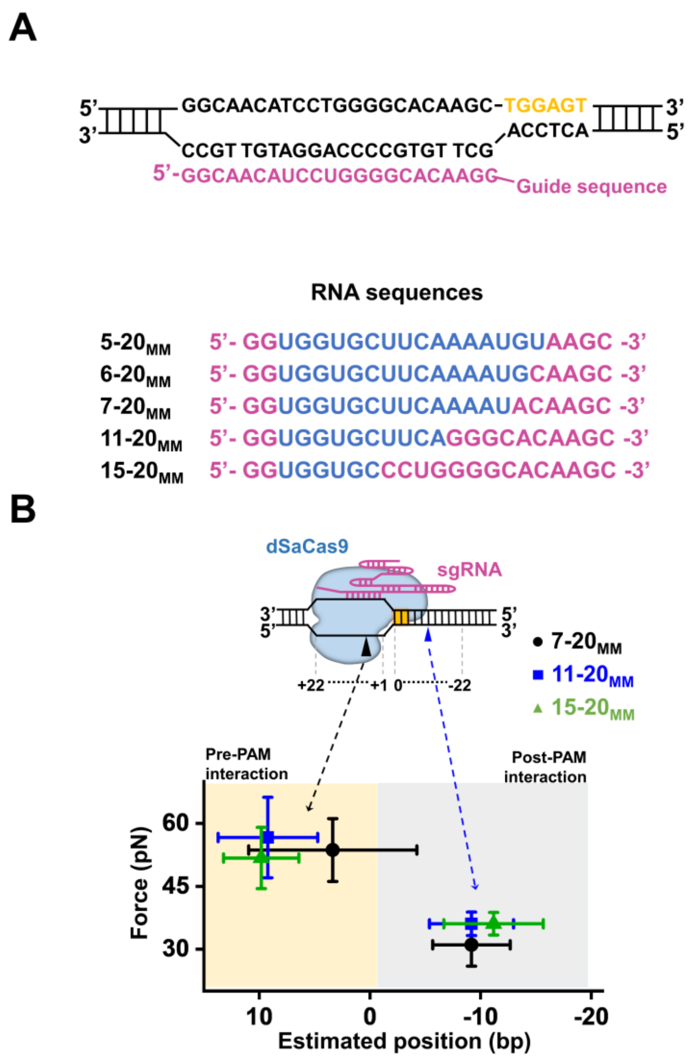

Appendix Fig S2 - Positions and disruption forces of the two interactions between dSaCas9 and DNA when guided by partially matched sgRNAs.

A. Schematic representation of sequences of DNA target and partially matched sgRNAs. The PAM is shown in yellow. The matched and mismatched sgRNA sequences are shown in purple and blue, respectively.

B. Positions and disruption forces of the two interactions between dSaCas9 and DNA when guided by mismatched sgRNAs. These data were obtained in the forward ($n_{7\text{-}20MM}$ = 14, $n_{11\text{-}20MM}$ = 25, $n_{15\text{-}20MM}$ = 26) and reverse ($n_{7\text{-}20MM}$ = 15, $n_{11\text{-}20MM}$ = 38, $n_{15\text{-}20MM}$ = 50) DNA unzipping experiments. The error bars represent the SD. The pre- and post-PAM interactions are conserved with these sgRNAs.

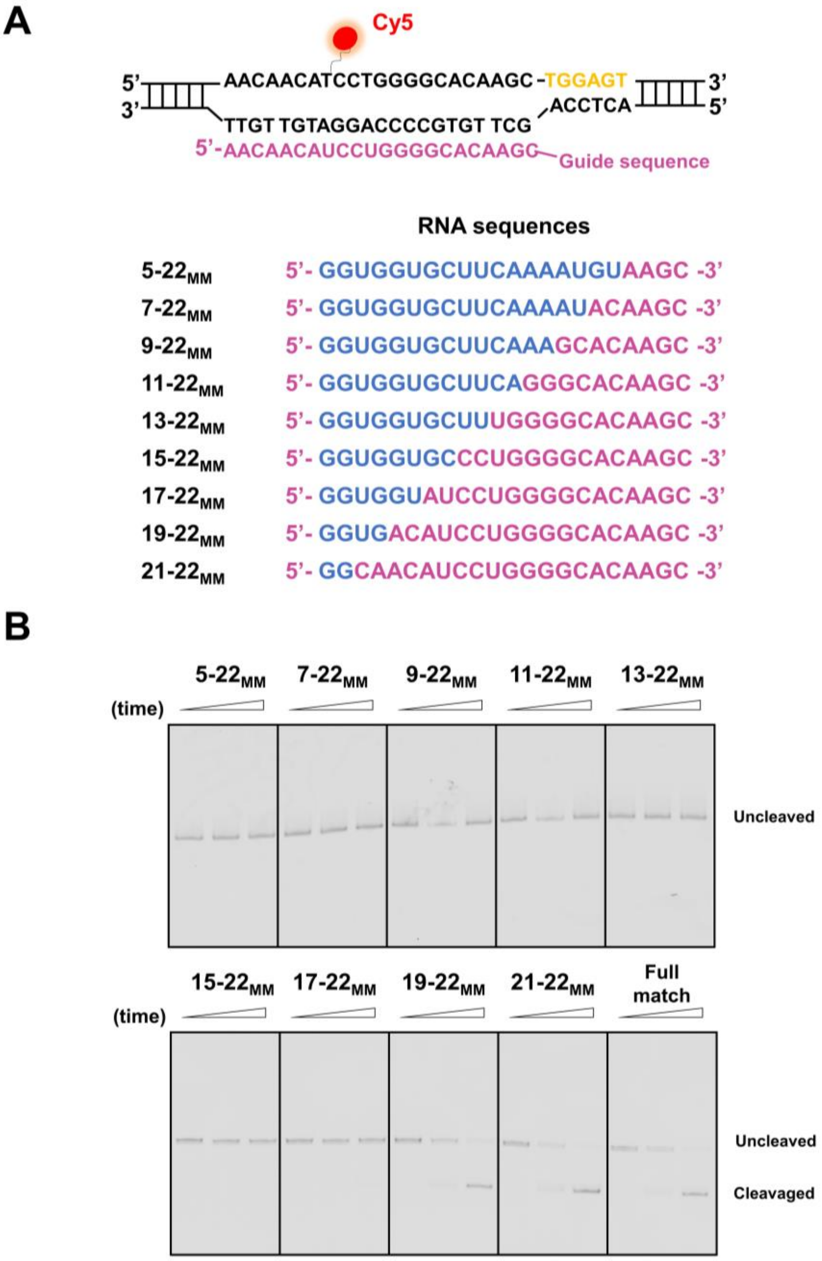

Appendix Fig S3 - DNA cleavage by SaCas9 when guided by PAM-distal mismatched sgRNAs.

A. Schematic representation of sequences of DNA target and guide RNAs. The PAM is highlighted in yellow. The matched and mismatched sgRNA sequences are shown in purple and blue, respectively.

B. DNA cleavage by SaCas9 guided by PAM-distal mismatched sgRNAs. The experiments were performed in triplicate and a representative gel image is shown. Eighteen RNA-DNA matches are required for SaCas9 to cleave the DNA target.

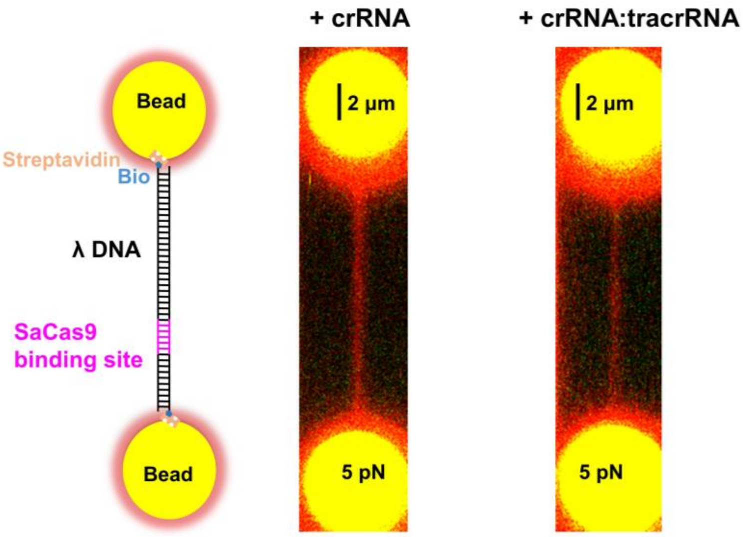

Appendix Fig S4 - Confocal image of the λ DNA in the presence of crRNA or crRNA:tracrRNA.

The λ DNA molecule was suspended between two microspheres held by two optical traps and was fluorescently labeled by TO-PRO-3. The 5’ end of crRNA was labeled with Cy3. A 5 pN stretching force was applied on the DNA molecule. In the presence of either crRNA (n = 13) or crRNA:tracrRNA (n = 11), fluorescent signal of the Cy3 fluorophore was not detected along the DNA template, indicating that the detected fluorescent signals in Fig 5C result from the binding of SaCas9 in complex with crRNA:tracrRNA on the DNA target.
