# Supplementary material for: Dynamics of Staphylococcus aureus Cas9 in DNA target Association and Dissociation
Source: EMBO Rep. 2020 Aug 13;21(10):e50184. doi: 10.15252/embr.202050184 (PMC7534634; doi:10.15252/embr.202050184)
Supplement: Supplementary file 2 — Expanded View Figures PDF [file EMBR-21-e50184-s002.pdf]

Expanded View Figures

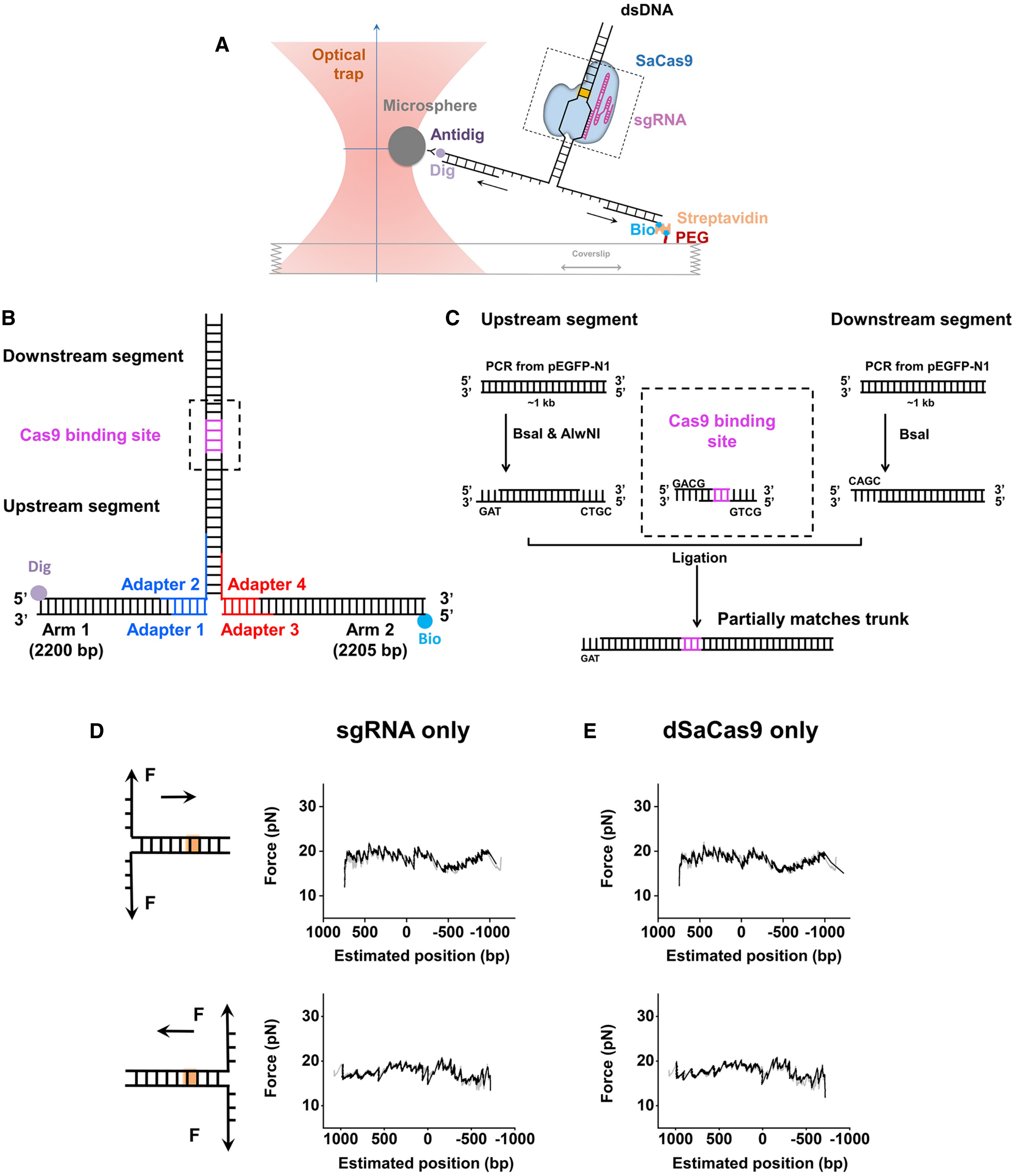

Figure EV1.

Figure EV1. Experimental configuration, DNA template design and representative traces in the presence of either dSaCas9 or sgRNA.

A Cartoon illustrating the single-molecule DNA unzipping experiment. One end of the two arms of the DNA construct was attached to a trapped microsphere and other end to the surface of a microscope coverslip. By moving the coverslip away from the trapped microsphere in one direction, dsDNA was sequentially converted to ssDNA upon base pair separation. As the unzipping fork progressed through SaCas9, it disrupted the interactions between SaCas9 and DNA at well-defined locations with distinct forces.

B The DNA construct was composed of three DNA segments—two arms and a trunk, which was linked through four short adapters (Table EV1). The trunk contained a single SaCas9-binding site (purple).

C Trunks containing partially matched DNA target site were ligation products of the following three DNA segments: the upstream segment, the SaCas9-binding segment and the downstream segment. Partially matched SaCas9-binding segments were generated by annealing two oligonucleotides (Table EV1).

D Representative DNA forward and reverse unzipping traces in the presence of sgRNA-1 showing the base pairs versus force. All DNA unzipping traces were comparable to that of naked DNA (gray), and no obvious rise in force was detected.

E Representative DNA forward and reverse unzipping traces in the presence of dSaCas9 showing the base pairs versus force. All DNA unzipping traces were comparable to that of naked DNA (gray), and no obvious rise in force was detected.

Figure EV2. Interactions between dSaCas9 and the DNA target.

A Histogram of the positions of the pre-PAM interactions detected from the forward DNA unzipping experiments.

B Histograms of the disruption forces of the pre-PAM interaction detected in the forward DNA unzipping experiments. DNA unzipping traces showing overstretching were counted as 65 pN and are indicated in red.

C The disruption forces measured in the forward DNA unzipping experiments under a wide range of unzipping speeds (n = 16, 27, 15, and 16 from left to right). The error bars represent the SD.

D Histograms of the positions of the pre-PAM (black) and post-PAM (blue) interactions detected from the reverse DNA unzipping assays

E Histograms of the disruption forces of the pre-PAM (black and red) and post-PAM (blue) interactions detected from the reverse DNA unzipping assays. DNA unzipping traces showing overstretching were counted as 65 pN and are indicated in red.

F The disruption forces measured in the reverse DNA unzipping experiments under a wide range of unzipping speeds (n = 19, 34, 19 and 19 from left to right). The error bars represent the SD.

G DNA-binding fractions of dSaCas9 after incubation with free DNA targets for the indicated number of hours at 37 °C ($n \geq 20$ for each condition).

 © 2020 The Authors

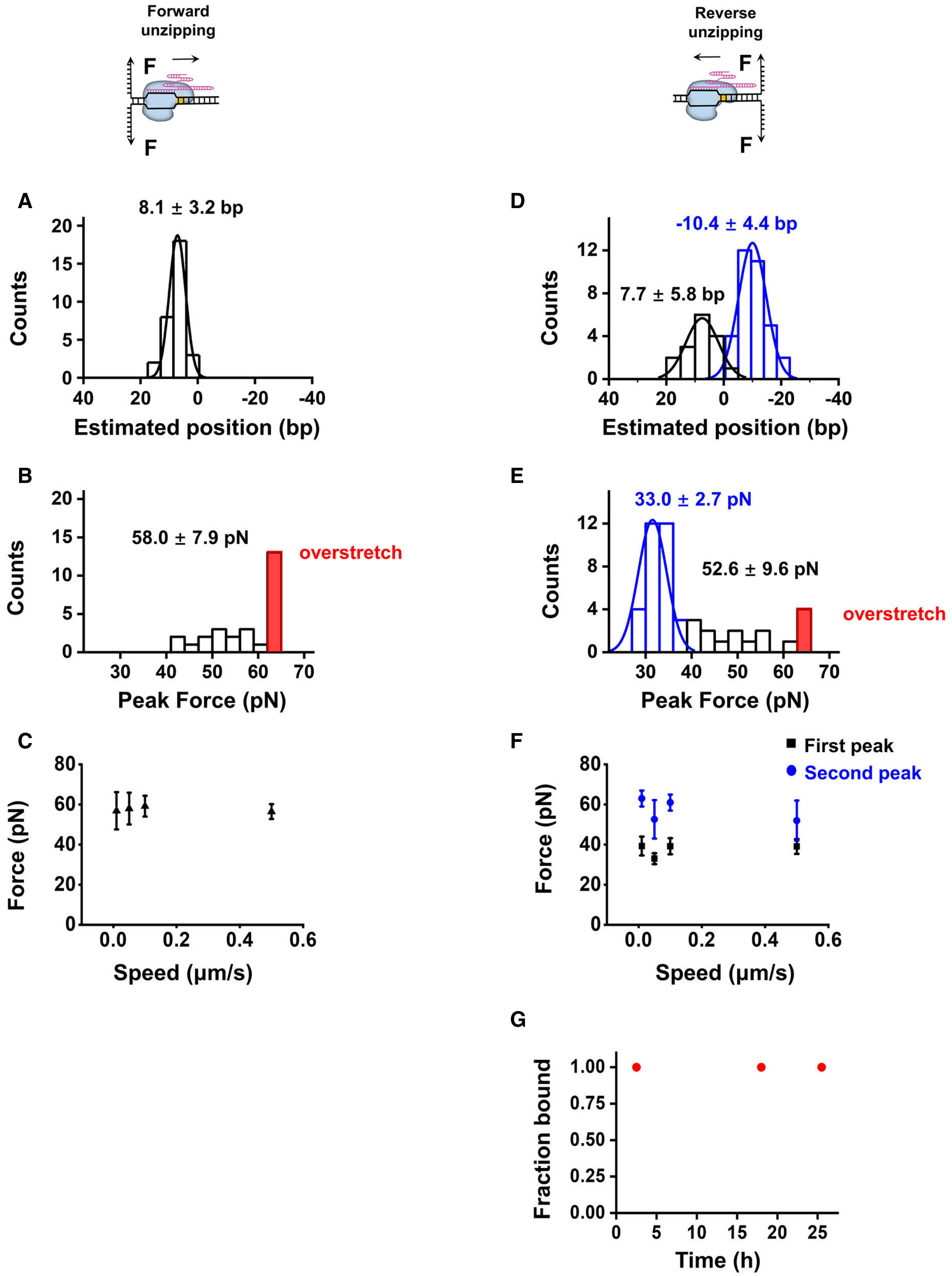
Forward unzipping
F
F
Reverse unzipping
F
F
A
8.1 ± 3.2 bp
Counts
Estimated position (bp)
B
58.0 ± 7.9 pN
overstretch
Counts
Peak Force (pN)
C
Force (pN)
Speed (μm/s)
D
-10.4 ± 4.4 bp
7.7 ± 5.8 bp
Counts
Estimated position (bp)
E
33.0 ± 2.7 pN
52.6 ± 9.6 pN
overstretch
Counts
Peak Force (pN)
F
First peak
Second peak
Force (pN)
Speed (μm/s)
G
Fraction bound
Time (h)

Figure EV2.

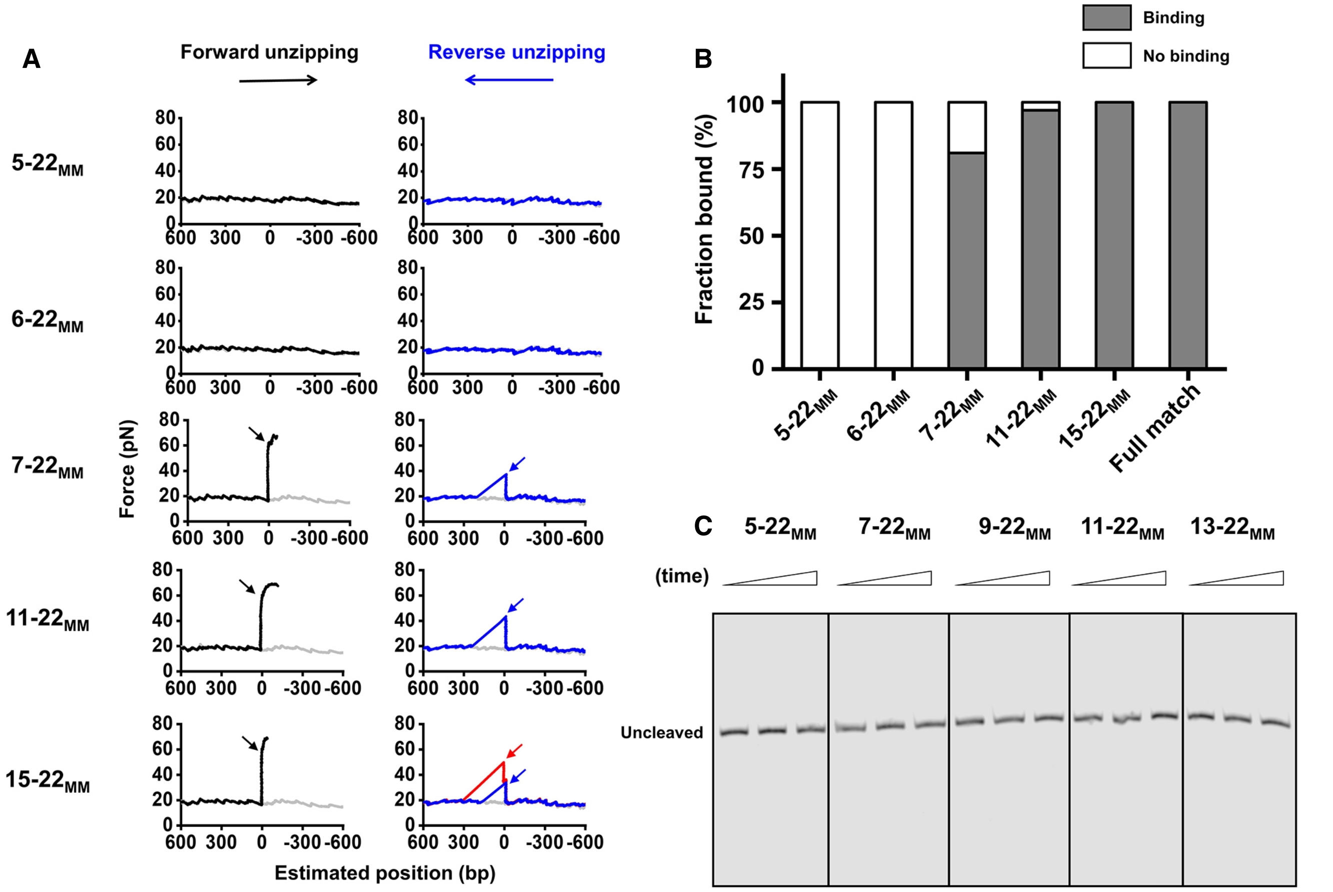

Figure EV3. DNA target binding and cleavage of SaCas9 with mismatched sgRNAs.

A Representative traces of the forward (black) and reverse (blue and red) mismatched DNAs (D5–22_{MM}, D6–22_{MM}, D7–22_{MM}, D11–20_{MM}, and D15–20_{MM}) unzipping in the presence of dSaCas9/sgRNA showing the force versus the number of unzipped base pairs. The naked DNA unzipping signatures are also presented for comparison (gray). The subscript "MM" represents the mismatch of the DNA sequence with the sgRNA-1. The black and red arrows indicate the pre-PAM interaction, and the blue arrows indicate the post-PAM interaction.

B The forward DNA unzipping assays revealed similar binding fractions of dSaCas9 to the DNA target with both fully matched (target) and mismatched sequences (n = 12, 13, 26, 42, 29, and 27 from left to right).

C DNA cleavage by SaCas9 guided by PAM-distal mismatched sgRNAs. Cleavage products were not detected with these five mismatched sgRNAs. The reactions were quenched at three time points (0, 15, and 60 min).

 © 2020 The Authors

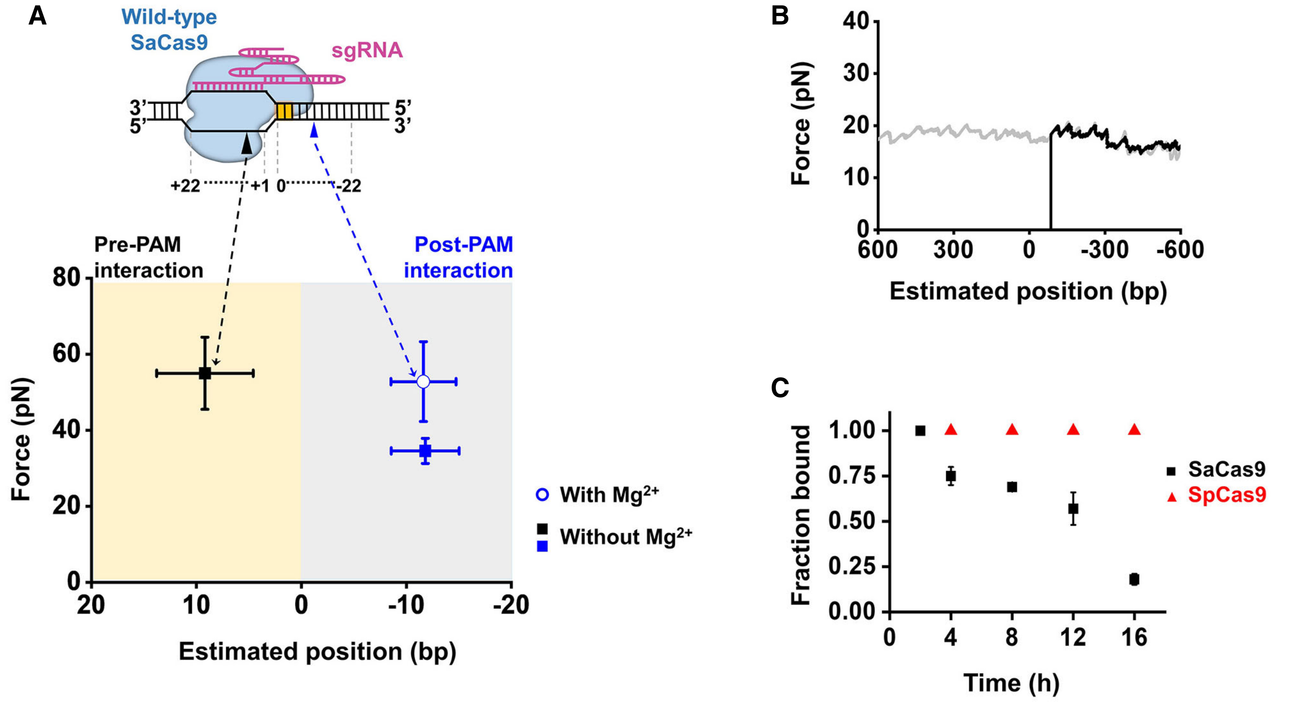

Figure EV4. DNA association and dissociation of wild-type SaCas9 and SpCas9.

A The forward and reverse DNA unzipping assays revealed the positions and disruption forces of SaCas9-DNA interactions in the presence (n = 24) and absence of Mg^{2+} (n = 37). Two interaction sites were detected with SaCas9 in the absence of Mg^{2+}, as a result of the incompetence of SaCas9 in DNA cleavage. In the presence of Mg^{2+}, a relatively stronger post-PAM interaction between SaCas9 and DNA was detected. The error bars represent the SD.

B Representative trace of the reverse DNA unzipping after incubation with SaCas9/sgRNA for 4 h showing the force versus the number of unzipped base pairs (n = 12).

C The fractions of SaCas9 (black square) and SpCas9 (blue triangle) bound to the DNA target after incubation with free DNA for the indicated number of hours at 37 °C. After incubating the target DNA with Cas9 at a 1:10 ratio for 10 min at 37 °C, excessive free DNA was added to serve as a trap for the indicated number of hours at 37 °C. DNA-binding fractions of Cas9 proteins were measured by the reverse DNA unzipping assays ($n \geq 20$ for each condition). The error bars represent the SD.
